# Supplementary material for: Patterns of Intron Gain and Loss in Fungi
Source: PLoS Biol. 2004 Nov 30;2(12):e422. doi: 10.1371/journal.pbio.0020422 (PMC532390; doi:10.1371/journal.pbio.0020422)
Supplement: Table S1 — Also available at http://genes.mit.edu/NielsenEtAl/. (4.3 MB ZIP). [file pbio.0020422.st001.zip › NielsenEtAl/html/800.html]

AN4295.1.NCU04307.1.MG00965.1.FG10319.1


```
 CLUSTAL W (1.82) Multiple Sequence Alignments - Introns Inserted


Sequence 1: NCU04307.1	198 aa
Sequence 2: FG10319.1	184 aa
Sequence 3: MG00965.1	187 aa
Sequence 4: AN4295.1	188 aa
Alignment Length: 201 aa
Number Identitical Residues: 103 aa
Alignment Score (without introns) 4908


MG00965.1 	MKVFTNKDTFNYSWEEVSTANWRKYCPWNDKSTHVIAVDTISRSVDSQTGI~LRTERLIT
NCU04307.1	MKVFSNTETFNYSWEEVSTANWRKYCPWNEKSTHVVAVDTISRSVDPATGI~LRTERLIT
FG10319.1 	MKVFSNSVTFNYSWDEVSTANWTKYGPWNNKSEHVIAVDTLSREVDPATGI~LRTERLIT
AN4295.1  	MKVFSSLCTFDYSWEEVSTANWRKYCPWNDKSTHVVGVDTLSRGVDPETGI0LRTERLIT
          	****:.  **:***:******* ** ***:** **:.***:** **. *** ********

MG00965.1 	CRQNAPDWVCKVLGASNGESLMYEVSYVDAANKKVTMVSQNMTWSNLINVQETVVYSPLN
NCU04307.1	CRQAAPEWLKSMMGGGMDESQVFETSYVDPKTKTVTMVSTNITWSNLLNVQETVVYSPLS
FG10319.1 	CKQSVPDWIKTIIGGTGDESFMYEASYVDPVNKTVTMVSQNLTWSNLVNVQEEVVYKPLG
AN4295.1  	CNQSVPQWVSSLFGGS-PTSHVYEVSYVDPISKKVTMCSTNLTWANVLNVRETVIYQPSV
          	*.* .*:*: .::*.    * ::*.****. .*.*** * *:**:*::**:* *:*.*  

MG00965.1 	DK---QTQFVQDAKITALCGGWQKIRNSIEDAIVTRFRENAAKGREGFESVLAMSRRVFA
NCU04307.1	DH---QTKFHQEAKITALAGGWQRIKNSIEDTLVKRFRENAAKGKEGFESVLAMSRRVFA
FG10319.1 	NH---QTQFIQNANITALCGGWQRIKNSIEDTMVSHFRENAVKGREGFERVLMMSRKAFA
AN4295.1  	TNPSSKTEFQQEAKITALCGGWQKIKNKVEEASVERFRENAKRGREGFEAVLEMSRRVFS
          	 :.:::*:* *:*:****.****:*:*.:*:: * :***** :*:**** ** ***:.*:

MG00965.1 	EERARQVVMAQ-----------
NCU04307.1	EEREREKMGLIREQAGNIRMAA
FG10319.1 	EERARQVL--------------
AN4295.1  	EQRALESSVA------------
          	*:*  :
```
